# Red Blood Cell Agglutination for Blood Typing Within Passive Microfluidic Biochips

**DOI:** 10.3390/ht7020010

**Published:** 2018-04-19

**Authors:** Maxime Huet, Myriam Cubizolles, Arnaud Buhot

**Affiliations:** 1 University Grenoble Alpes, F-38000 Grenoble, France; m.maxime.huet@gmail.com (M.H.); myriam.cubizolles@cea.fr (M.C.); 2 CEA LETI MlNATEC Campus, F-38054 Grenoble, France; 3 University Grenoble Alpes, CEA, CNRS, INAC, SyMMES, F-38000 Grenoble, France

**Keywords:** quantitative agglutination assay, passive microfluidic biochip, embedded reagents, automated image processing, real time detection, blood typing, Point-of-Care

## Abstract

Pre-transfusion bedside compatibility test is mandatory to check that the donor and the recipient present compatible groups before any transfusion is performed. Although blood typing devices are present on the market, they still suffer from various drawbacks, like results that are based on naked-eye observation or difficulties in blood handling and process automation. In this study, we addressed the development of a red blood cells (RBC) agglutination assay for point-of-care blood typing. An injection molded microfluidic chip that is designed to enhance capillary flow contained anti-A or anti-B dried reagents inside its microchannel. The only blood handling step in the assay protocol consisted in the deposit of a blood drop at the tip of the biochip, and imaging was then achieved. The embedded reagents were able to trigger RBC agglutination in situ, allowing for us to monitor in real time the whole process. An image processing algorithm was developed on diluted bloods to compute real-time agglutination indicator and was further validated on undiluted blood. Through this proof of concept, we achieved efficient, automated, real time, and quantitative measurement of agglutination inside a passive biochip for blood typing which could be further generalized to blood biomarker detection and quantification.

## 1. Introduction

Among a wide range of characteristics or phenotypes, the blood of an individual is categorized by its group that is defined by the presence or absence of specific antigens at cell’s surfaces and specific antibodies in the serum. Along with the discovery of new antigens, new blood grouping systems are created. However, the most generally known are the ABO system categorizing bloods as group A, B, AB, or O. In addition, the rhesus (Rh) system, which is indicated by the sign + or −, is representative of the presence or absence of the D antigen at the red blood cells (RBC) surface [1].

The most frequent need for the knowledge of someone´s blood group concerns blood transfusion and organ transplant [2]. Although there are now techniques to achieve ABO-incompatible organ transplantation [3] for blood transfusion, ABO compatibility test between a donor and his recipient is mandatory [4]. If the donor and the recipient have incompatible groups, a transfusion reaction occurs and it could lead to acute and fatal hemolysis [5]. The most frequently cited causes of error are human mistakes related to management or organization of the transfusion chain [5,6,7]. This is the main reason why an ultimate bedside ABO assay is performed just before the transfusion: the pre-transfusion bedside compatibility test (PBCT). 

Although an ultimate control before transfusion must be performed, a recent study of 26 lethal accidents due to ABO incompatibility [8] showed that, in 32% of the cases, PBCT was not done at all, in 24%, the PBCT was not done at the patient bedside, and in 44%, it was done only on the transfused unit and not on the patient. Lack of ease of handling, the need of extra materials (equipment, solutions…), and the time that is required to perform the analysis restrain the proper use of PBCT [9]. Thus, an improper use of PBCT rendered its use almost totally inefficient as errors might still occur before transfusion. Furthermore, when the PBCT was performed, in 20% of the previously mentioned accidents, the results were either not correctly interpreted, found to be not interpretable, or not taken into account. Another study of two commercial bedside ABO compatibility tests showed that errors occurred in 30% of the assays performed [10]. The errors were attributed to poor technique of the operator, device failure, or incorrect interpretation. Finally, the protection of the operator [11] and the cost of the PBCT device [12] were also important criteria to consider. 

ABO group of an individual may be determined through two different sets of techniques. Firstly, genotyping techniques are used to determine the genotype before predicting the phenotype [13]. One notable application of those DNA-based methods is the determination of fetal blood group in pregnancies when the fetus is at risk for hemolytic disease of the fetus and newborn [13]. DNA based blood typing is also used in forensic science to match bloodstains at crime scene with other biological samples that are not blood [14,15,16]. Those genotyping methods suffer from the discrepancy between ABO genotype and phenotype in some cases [17], as the gene does not code for the antigen itself, but for an enzyme catalyzing the reaction setting the type A, B, or O of the RBC antigens. Furthermore, such methods are not fully adapted to PBCT in terms of equipment, cost, and time that is required for the analysis. Secondly, the phenotype may be directly determined using hemagglutination techniques. Two different procedures exist either forward or reverse typing [18]. The presence or absence of antigens at cell’s surface determination is called forward typing, while the determination of the presence or absence of antibodies in the plasma is named reverse typing. We did not consider reverse typing, since it is not performed regularly for the ultimate control at the patient bedside in France [12]. On the contrary, the forward typing is commonly used by hemagglutination on different format: a microplate [19], a plate or a tube [9], or a gel column [20]. However, these techniques generally require centrifugation, the use of liquid reagents, and often manual agitation. The lateral flow assays are also a method of choice for the multi-parameter identification of blood groups [21], but the protocol usually requires several handling steps under liquid format (blood dilution, mixing, washing). 

The present study addressed the real-time observation and the quantitative measurement of agglutination in passive microfluidic biochips. ABO forward blood typing by hemagglutination has been performed as a proof of concept on whole blood, while the previous study focused on 1:5 diluted blood [22]. The principal drawbacks of the existing techniques were addressed. Thus, the proposed approach aimed at a low cost, rapid, and sensitive analysis that was easy to use with good operator safety, and that was able to quantitatively and automatically assess the agglutination. To prevent liquid reagent manipulation, the reagent triggering the agglutination was embedded inside a microfluidic chip and a passive blood filling was ensured by capillary flow. The spontaneous capillary flow prevented accidental blood projections that were induced by forced flow actuation, reducing the contamination risk for the human operator. The microfluidic chip design required no assembly steps and was compatible with rapid industrial fabrication at low cost by injection molding. An optical instrument and an image processing algorithm allowed for us, respectively, to obtain the real time observation and for the automated computation of the agglutination results. Thus, quantitative agglutination criteria were defined and optimized with a first training set on diluted bloods. The methodology was developed with two different algorithms that were based on variance and correlation of images for 1:5 diluted blood [22]. The agglutination of diluted RBC presented important modifications on the local density which impacted significantly the images. The case of undiluted blood is more complex since RBC are nearly touching each other since they represent a large fraction of the volume. It was thus necessary to confirm that the methodology that was previously developed was still efficient for undiluted blood. Thus, validation sets using blood samples from different patients and various biochip batches aimed at confirming the quantitative agglutination criteria on diluted and undiluted bloods. Their validation confirmed the excellent sensitivity and specificity of this real time quantitative approach for ABO blood typing in the case of correlation quantification. Those quantitative agglutination criteria could offer a strong help in the bedside operator decision, therefore improving from the generally used naked eye qualitative criteria requiring experienced operators. Furthermore, the real time observation of the agglutination will open the door to a large set of potential applications besides ABO blood typing, like biomarkers detection and quantification by hemagglutination or even latex agglutination assays [23].

## 2. Materials and Methods

### 2.1. Microfluidic Chip 

The design of the microfluidic chip was optimized for passive injection of blood [24]. It is fabricated by the injection molding of cyclic olefin polymer (COP) (Cema, Le Mans, France). The wettability of the COP was increased by exposing the microfluidic chip to O_2_ plasma using a MVD100 (Applied Microstructures, San Jose, CA, USA) during 10 min at a flow rate of 450 cm^3^·min^−1^ and 200 W power. The hydrophilic treatment was checked by measuring the contact angle of water on the COP. The measure was performed using a DSA100 (Krüss, Germany) by deposing a 2.5 µL drop of water on the elbow part of the microfluidic chip.

### 2.2. Embedded Reagents 

Anti-A and anti-B blood typing reagents were obtained from GROUPAKIT (Diagast, Loos, France) and are specific for blood typing. These two liquid reagents were reformulated by Avalun (Grenoble, France). Those solutions were separately embedded in microfluidic chips by drying to make anti-A biochips or anti-B biochips. The drying process was achieved overnight at room temperature in a vacuum desiccator (DURAN, Mainz, Germany), all of the biochips were held firmly and totally filled by 6.5 µL of solution [22]. The biochips were then stored in the dark at room temperature before use.

### 2.3. Blood Samples 

Blood samples were obtained from healthy donors (Etablissement Français du Sang (EFS), Grenoble, France) and were collected in EDTA vacutainer tubes (Becton Dickinson, Le Pont de Claix, France). They were used either after 1:5 dilution with PBS (Sigma-Aldrich, Steinheim, Germany) or without any dilution. In any case, an informed consent was given by blood donors according to the ethical and legal standards of our blood supplier (EFS). The blood tubes were delivered three days after withdrawal and were kept at 4 °C in a fridge for storage. The experiments were performed within three days after delivery. The time delay between collection of the blood and its analysis may affect the rheological behavior of the blood with the formation of aggregates of RBC or rouleaux [25]. However, the image processing algorithm was developed from the training set and was confirmed on the validation sets that were able to distinguish aggregation (rouleaux formation) from agglutination. Before any experiments, 500 µL of each blood samples were pre-warmed at room temperature (25 °C). The temperature of the blood inside the biochip was measured with a thermocouple (ThermoCoax, Suresnes, France) and never exceeded 27 °C when exposed to the microscope lamp for the whole duration of a blood typing test.

As a routine procedure, EFS determined the blood group of the donors. Those results were used as controls for our blood typing tests. A first set of 24 experiments were performed on 1:5 diluted bloods and were used as an optimization set to determine the parameters for the calculation of the agglutination indicators [22]. For the optimization set, blood samples from four different donors were selected: two of A blood group, one of B blood group, and one of O blood group. Then, two validation sets were studied. The first validation set of eight experiments with 1:5 diluted bloods was used to check that the selected parameters were still relevant to discriminate positive and negative agglutination using the indicators. The second validation set of 10 experiments on undiluted bloods served to adjust the parameters to undiluted blood and to validate the method in real conditions for point-of-care (POC) applications. For the first validation set, blood samples from two donors were selected, one is A the other is O, while for the second validation test, the two donors were from group A and B, respectively. Optimization and validation sets were performed at several months distance using different blood samples and biochips from different batches of embedded reagents to assess the reproducibility and the robustness of the method.

### 2.4. Operating Protocol for the Blood Typing Experiments 

**Figure 1:**
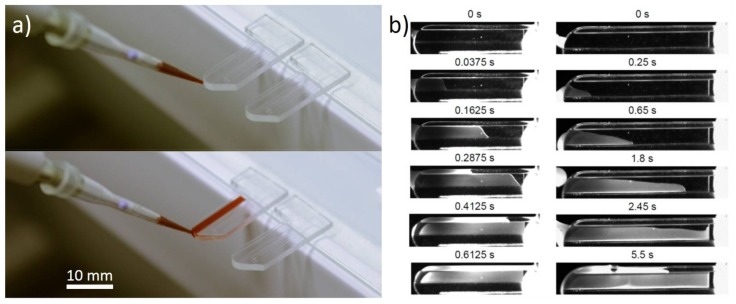
(**a**) Blood injection inside (unembedded reagents) biochips; (**b**) Kinetics of blood draining inside biochips without embedded reagents (left) and with embedded reagents (right). Timescale of the different images are indicated above.

For better handling with the optical microscope, the non-functional end of the biochip was stuck with double sided tape on a microscope glass slide (Figure 1a). The blood was maintained at room temperature (25 °C) using a Thermomixer. A volume of 6.5 µL of 1:5 diluted or undiluted blood was deposited using a P10 micropipette (Eppendorf, Hamburg, Germany) at the curved tip of the biochip (illustration in Figure 1a). The blood filled the channel of the biochip by passive capillary forces [24]. Draining kinetics were affected by the presence of embedded reagents inside the biochips. While the blood filled the biochips in less than one second without embedded reagents, biochips containing an anti-A or anti-B reagent were filled in 5 to 6 seconds without affecting the agglutination process. 

### 2.5. Observations, Video Recording 

Observations were made with an optical upright microscope, Olympus BX60 (Olympus, Shinjuku, Tokyo, Japan) with an X5 objective lens. The transmitted light was recorded by a monochrome camera Mightex BTE-B050-U (Mightex, Toronto, ON, Canada) in 8 bits mode. For 1:5 diluted bloods, the pictures were recorded using 1:2 decimation, leading to a resulting resolution of 1296 × 926 pixels. For undiluted bloods, no decimation was performed to keep the best resolution of the images. For each experiment, an image sequence was recorded during two minutes at one frame per second. The recording started three to ten seconds before the blood was deposited at the tip of the biochip with the initial time t = 0 s set at the beginning of the acquisition. Thus, when considering the 5 to 6 s that is necessary for injection of the blood inside the biochips, the images before the first 20 s were discarded to avoid incorrect image processing. The blood filling inside the biochips by spontaneous capillary flow was made through video recording by the same Mightex camera at 40 frames per second and with a 5–40 mm C-mount objective (COMPUTAR, Tokyo, Japan). Blood filling was compared on two kinds of biochips: a biochip containing embedded reagents and an empty microfluidic biochip to analyze the impact of the dry reagents on the passive flow (Figure 1b). 

### 2.6. Image Processing and Agglutination Indicator Computation

An image processing algorithm was developed using Matlab R2013a (MathWorks, Natick, MA, USA) to automatically and quantitatively measure the agglutination [22]. This agglutination quantification relied on a correlation indicator between different pixel lines. Kinetic results were achieved by computing the indicators for every image. 

The image processing was performed on the whole picture length within a main region of interest (ROI) of 50 pixels in height for 1:5 diluted bloods and 800 pixels in height for undiluted bloods starting at the line 101 from the V-groove. The first hundred lines have been removed to avoid artifacts due to the edges. The correlation indicator was determined as the correlation between lines distant from five pixels in height for 1:5 diluted bloods and 12 pixels for undiluted bloods. Due to the 1:2 decimation for diluted blood experiments, the 12 pixels for undiluted blood would correspond to six pixels with 1:2 decimation. The main ROI was divided into 16 sub-ROI and the agglutination indicators were computed for each sub-ROI allowing for the assessment of their variability (see [22] for more details). 

## 3. Results

### 3.1. Microfluidic Biochip Design and Fabrication

The microfluidic biochip was optimized with a simple design consisting in an open straight channel forming a V-groove at the bottom to enhance filling speed by capillary flow. Hence, no fluid actuator was required, which was a major advantage for POC use when compared to other methods [4,18]. Some studies comparing similar overall design with and without the micromixer revealed that the micromixer was necessary to detect agglutination [4]. In our approach, complex fluidic functions, like micromixer, were not required for a quantitative measurement of agglutination. Other studies using more complex fluidic functions needed assembly steps to seal the channel due to the use of fluid actuators [18], leading to an increase in cost and fabrication time. Otherwise, it was also possible to use more sophisticated fabrication techniques, like micro-stereo-lithography, to avoid the assembly steps [4], however, at the cost of a dramatic increase in the fabrication time (13 h in this example). Our open design was compatible with quick fabrication and at low cost by injection molding techniques without any assembly step. Thus, it reduced the potential problems with tubing (biofouling, sterility, dead volume, leakage…), power consumption, handling difficulty, and the need for bulky hardware.

### 3.2. Capillarity Filling of the Channel

To ensure the perfect filling of the biochip by the blood, an O_2_ plasma treatment of the COP polymer was necessary to enhance the hydrophilicity. Before exposure to O_2_ plasma, the water contact angle of the COP was measured at 94 ± 1° (mean value ± standard deviation), and after the treatment it was reduced to 33 ± 7°. Thus, the plasma effectively increased the hydrophilicity of the channel of the biochips and led to a blood drop of 6.5 µL successfully filling the microchannel that was not embedded with any dried reagent in less than 1 second (Figure 1b). The embedding of reagents inside the channel affected the capillarity draining forces increasing the filling time of the whole biochip containing the embedded reagent in 5 to 6 s (Figure 1b).

### 3.3. Image Observation of the Agglutination

**Figure 2:**
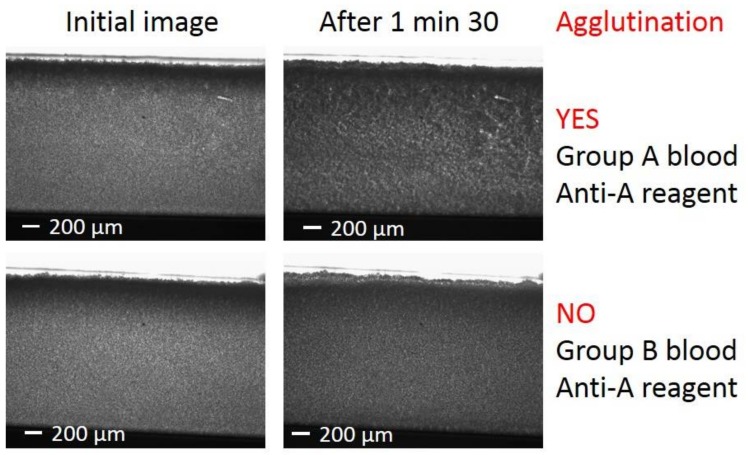
Microscope images: (**Left**) Initial images just after the entrance of the blood inside the microfluidic biochips. (**Right**) Images taken 90 s after the injection of the blood. (**Top**) Positive agglutination: images for group A blood injection in anti-A reagent biochip. (**Bottom**) Negative agglutination: images for group B blood injection in anti-A reagent biochip.

A first simple direct interpretation of the agglutination state of the blood in the biochip for each experiment may be performed when looking at the last pictures of each images sequence. The initial images just after injection of group A and group B bloods in biochips that were embedded with anti-A reagents looked alike (Figure 2 left). However, after 90 s, the acquired images were clearly different (Figure 2 right). While images at different times were similar for the group B blood with anti-A reagents, for blood A group, a clear evidence of agglutination existed. This distinction between A and B blood groups confirmed that, at least partial, activity recovery of the dried antibodies was ensured after the dissolution in the blood samples. In certain cases, the interpretation of the agglutination results was not straightforward from simple naked-eye observation, which required the use of an image processing to unambiguously determine the agglutination state. Furthermore, the important delay between blood collection and analysis could alter the rheological properties of the blood due to the formation of aggregates of RBC or rouleaux similar in shape to agglutinates. Fortunately, the formation of rouleaux inside the biochips has a negligible effect when compared to agglutination on the image processing algorithm, thus, the agglutination process was not masked by RBC aggregates. 

### 3.4. Choice and Optimization of the Agglutination Indicator

The binding of RBCs by anti-A or anti-B IgM antibodies interacting with the corresponding antigens at their surface lead to agglutination. The number of bonds between the RBCs increased with time leading to a change in the spatial distribution of cells in the biochips and thus of the light absorption as observed by the optical microscope. Higher density zones appeared and got densified along the time while at the same time zones of lighter density, and thus smaller absorption appeared. The consequences are a spreading of the gray level distribution hence an increase in the variance. Thus, the first choice of agglutination indicator was based on variance [22]. Although, it was sufficient to discriminate positive and negative agglutinations for the diluted blood samples, it was not efficient for undiluted ones. Furthermore, such a variance indicator did not take into account the spatial information of the RBC aggregates from the images, hence, a second agglutination indicator was considered that was based on correlation [22]. Such an agglutination indicator appeared to be efficient for diluted as well as undiluted blood samples.

During an agglutination, RBCs undergo an evolution from randomly distributed cells to growing clusters or aggregates. Thus, growing areas of similar gray level appeared on the images and the neighboring pixels got more correlated. For the correlation based agglutination indicator, it was chosen to compare pairs of lines instead of a kernel scanning its neighborhood in all of the directions for the sake of simplicity of the implementation and for the reduced computational time. The determination of the optimum parameters for the correlation agglutination indicator was optimized on the best discrimination between positive and negative agglutination after 90 s of blood sample injection on the optimization set. The parameters that were considered were the number of lines considered for the calculation, as well as the distance from the two correlated lines. For 1:5 diluted blood samples, 50 lines were sufficient for a perfect discrimination with an optimum found for a separation of five lines for the correlation [22]. 

**Figure 3:**
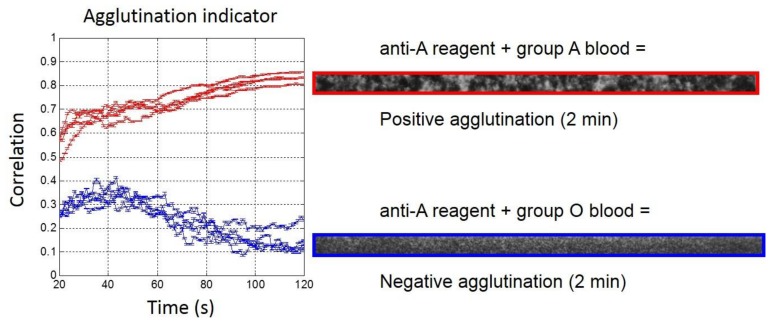
(**Left**) Real-time agglutination indicator measurement for the eight experiments of the validation set with 1:5 diluted bloods (Red: Group A and Blue: Group O) for anti-A reagent biochips. (**Right**) Agglutination image observed after 2 min on the region of interest (ROI).

Analysis of the validation set with 1:5 diluted blood samples (Figure 3) confirmed that the quantitative measurement of agglutination was already possible after 20 s and for the full length of the observation (up to 2 min). The agglutination indicator was always higher than 0.4 when agglutination occurred, while without agglutination, it remained below 0.4, with an over-shoot that was close to 0.4 after 40 s of blood injection. 

**Figure 4:**
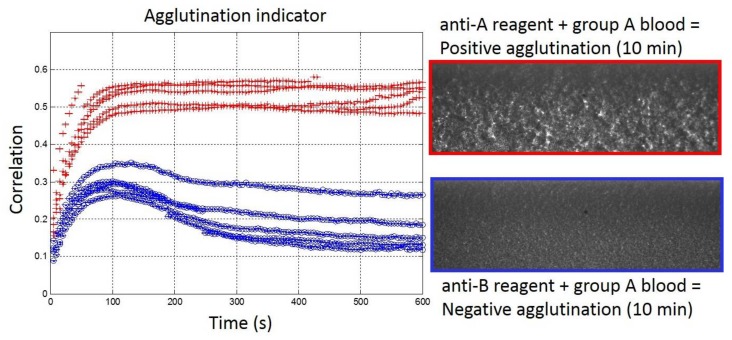
(**Left**) Real-time agglutination indicator measurement for the ten experiments of the validation set with undiluted bloods (Red: Group A and Blue: Group B) for anti-A reagent biochips. (**Right**) Agglutination image observed after 10 min.

The results of the validation set with undiluted blood samples are represented in Figure 4. As mentioned in the Materials and Methods, the parameters for the correlation indicator were modified for undiluted blood samples when compared to the optimized ones for 1:5 diluted blood samples. In particular, the number of lines that were considered for the determination of the correlation indicator was enlarged from 50 to 800 lines to ensure the correct quantification of the agglutination. Furthermore, the correlated lines were separated from five pixels for 1:5 diluted blood samples with 1:2 decimation, which would correspond to 10 pixels without decimation. In fact, a separation of 12 pixels between the correlated lines was necessary to ensure perfect discrimination between samples with or without agglutination. With those new parameters, the second validation sets confirmed the determination of blood groups in consistency with EFS results (Figure 4). The threshold value of 0.4 for the determination of positive/negative agglutinations remained relevant. However, due to undiluted blood and the large amount of RBC in the samples, the agglutination process was slowed down. It appeared that the indicator reached the threshold of 0.4 around 50 s after the blood injection. In comparison, with 1:5 diluted blood samples, the indicator was already above 0.4 after 20 s. Furthermore, with undiluted blood samples, the stabilization of the agglutination indicator occurred only after around 10 min, while 100 s were sufficient with 1:5 diluted blood samples. Interestingly, the over-shoot observed around 0.4 after 50 s with 1:5 diluted blood was still present with undiluted blood but shifted to lower values (around 0.3) and at later times (around 100 s). Unfortunately, the saturating value of the indicator in the presence of positive agglutination was also reduced for undiluted blood (around 0.5) compared to 1:5 diluted blood (around 0.8), thus, reducing the range of discrimination at the end of the agglutination process between positive and negative agglutination states. Finally, with slightly adapted parameters, we have shown that the real-time and quantitative measurement of the agglutination process was possible within our embedded reagents passive biochips in less than 10 min on undiluted blood samples for POC applications. 

## 4. Discussion

In this work, we addressed ABO forward blood typing by quantifying the agglutination state inside a passive microfluidic biochip. In the context of ABO assays that were performed just before transfusion several specifications, POC analyses are of paramount importance in order to get a high-quality ultimate control at the patient bedside [26].

### 4.1. Ease of Handling

Firstly, the ease of handling of the device is requested to prevent at maximum human errors [12]. Ideally, a perfect device would only request a blood drop deposit. This point was particularly well addressed by Li et al. [27]. Their device required to simply add a blood droplet inside the channels that were containing embedded reagents. Typically, it took 1 min for a blood volume of 3 µL to spread and to dissolve completely the antibodies before to get the results of agglutination state by naked-eye observation. Another study presented also the advantages of a simple protocol [28]: ABO and Rh determination were achieved within 2 min using 2 µL of whole blood that was spread directly onto dried reagents that were embedded in threads. Our work compared well to these previous studies, since we have chosen to use a passive microfluidic device containing dried IgM reagents requesting no actuation [23,29], nor active micromixer [18], nor (di)electrophoresis [30] to trigger in vitro agglutination. Indeed, our passive biochips containing embedded reagents did not require any human intervention after the deposit of the blood sample. The agglutination assay was completely autonomous, and was thus not subjected to human mistakes. 

### 4.2. Cost of Analysis

Another important criterion concerns the cost of the analysis. In the perspective of a POC routine blood typing analysis, the cost-effective fabrication process was mandatory for the microfluidic biochip fabrication. Good candidates for such manufacturing were injection molding [18] and embossing, allowing for the production of a huge number of plastic consumables for a reasonable cost [31]. Other alternatives can be threads [28], strips [32], or paper substrates [33,34,35,36,37]. By using a single microfluidic component that was obtained directly by injection molding without any additional assembly [38], we addressed the cost production issue. 

### 4.3. Operator Safety

Operator safety is also an important parameter to consider. Depending on the substrate materials or the protocols that were used, blood can overflow or leak from the system and get in contact with the operator. This is particularly the case when fluid actuation is mediated by positive pressure [31], or when the protocol implies several steps, such as sampling, incubation, and pipetting [37]. We attempted to simplify at maximum the agglutination protocol steps in order to avoid any useless contact of the operator with the blood patient. By using a passive microfluidic biochip without any actuation, we minimized the risk of the operator to be exposed to overflowed blood. 

### 4.4. Results Reading

In the context of POC, naked eye observation and the interpretation of the results is often a method of choice [18,27,31,39]. Nevertheless, as reported in previous studies, the risk of human errors is not negligible [40,41]. To limit the risk of human error in reading the result of the assay, a smart solution was presented by Li et al. [35], it consisted in writing the blood group results owing to the embedded reagents and ink. Some published data reported the use of the automated detection of agglutination by the absorbance measurement [42], by contrast image analysis [29], or by algorithms of classification [43]. However, most of these studies rely on end point detection. Real time detection of ABO blood typing was demonstrated by surface plasmon resonance detection in recent studies [44,45], but without measuring an agglutination of RBC. In our method, we developed an automated data analysis to follow in real time the agglutination process. Based on a fully automated process of image analysis, we used an agglutination indicator, which was based on correlations, in order to achieve a good reliability of the method and to ensure the robustness of agglutination state results. This will provide a quantitative help in decision making and traceability of the performed assay when connected to the hospital information management system.

## 5. Conclusions

In conclusion, we have presented an original method for the real time observation and the quantitative measurement of agglutination in passive biochips with a simple protocol on undiluted blood samples. Thus, we successfully designed an integrated blood typing test that did not require operator intervention, except for deposit of the blood drop (only 6.5 µL). No complex fluidics functions, like mixing nor equipment for fluid actuation, were used here. The blood typing test required a positive or a negative result: agglutination indicators fulfilled this need with a 100% success rate in less than ten minutes. To do so, a bulky imaging system and a computer to run the algorithm were presently necessary to complete the test. For an ultimate bedside ABO control before transfusion, the present work would benefit from investigation on hardware and software integrations. In order to analyze in detail the sensibility and the specificity of our approach, a larger set of blood samples should be considered, including various types of patients (age groups, gender, blood groups...). Further investigation on Rh typing and specific samples, like weakly interacting blood groups, including A3 (subgroups of A), B3 (subgroups of B), and patients with hematologic malignancy or thalassemia [38], should also be tested. For real ABO and Rh blood typing applications, multiplex approaches are possible with dedicated multi-channels biochips or multi-biochips by a single reading device. This could open the door for a multiplex device allowing for both forward and reverse blood typing being performed simultaneously. 

We demonstrated the feasibility of our method by performing ABO blood typing as a proof of concept, but our approach opens huge perspectives to other kind of agglutination assays. Indeed, we have recently shown that, by using bispecific reagents, it was possible to extend the use of agglutination of RBC to the detection of biomarkers [46]. Furthermore, the real-time observation and the quantitative measurement of the agglutination is sufficiently general to be generalized to latex or magnetic beads agglutination assays [47,48].
